# CA 15 − 3 may be a promising biomarker for differentiating distant organ metastasis in breast cancer at initial diagnosis

**DOI:** 10.1186/s12885-025-15463-4

**Published:** 2025-12-15

**Authors:** Serkan Yilmaz, Mesut Yur

**Affiliations:** 1 Department of Surgical Oncology, Faculty of Medicine, Fırat University, Elazığ, 23200 Turkey; 2 Department of Surgical Oncology, Faculty of Medicine, Yahya Kemal Street, Fırat University Hospital, Elazığ, 23200 Turkey

**Keywords:** Breast cancer, Metastasis, CA 15 − 3, CEA, Biomarker

## Abstract

**Background:**

Imaging techniques for breast cancer identify distant organ metastasis, but can be time-consuming and costly. This study compares clinicopathological and laboratory data between metastatic and nonmetastatic breast cancer patients at initial diagnosis and evaluates CA 15 − 3’s predictive value for distinguishing distant metastases.

**Methods:**

This retrospective study examined 232 untreated breast cancer patients diagnosed between January 2021 and January 2022. They were divided into distant organ metastatic (Group I) and nonmetastatic (Group II) at the initial diagnosis. We analyzed laboratory data, including CA 15 − 3 and CEA levels, and used Receiver Operating Characteristic (ROC) curves to find optimal cut-off values for predicting distant organ metastasis.

**Results:**

Forty-nine patients (21.1%) had distant organ metastases at initial diagnosis. Significant differences were found between groups in tumor diameter, neutrophil and lymphocyte counts, T-stage, N-stage, CA 15 − 3, CEA, albumin, alkaline phosphatase, neutrophil-to-lymphocyte ratio, and the systemic immune inflammation index (SII) (all *p* < 0.05). CA 15 − 3 demonstrated excellent discriminatory ability (AUC = 0.950; 95% CI: 0.924–0.975; *p* < 0.001). A CA 15 − 3 cutoff value of 25.8 U/mL had 100% sensitivity, 82.5% specificity, 60.5% positive predictive value, and 100% negative predictive value. No patient with a CA 15 − 3 level below 25.8 U/mL presented with distant organ metastasis.

**Conclusions:**

CA 15 − 3 demonstrated promising performance in ruling out distant metastases in newly diagnosed breast cancer patients. Further prospective studies are needed to confirm whether using CA 15 − 3 may reduce the time loss and costs associated with routine imaging for distant organ metastasis.

## Introduction

Breast cancer is the most common type of cancer in women and the second leading cause of death. It is estimated that approximately over 310,000 new cases of breast cancer will be diagnosed in 2024 [1]. Due to its high prevalence and association with death, early diagnosis has come to the forefront. Screening mammography for early diagnosis and initiating early screening for high-risk individuals via breast ultrasonography (USG) or magnetic resonance imaging (MRI) are both practices that are carried out [2]. Although the rate of early diagnosis has increased, metastasis to distant organs is still present at diagnosis.

Breast cancer is a systemic disease whose treatment is coordinated by a multidisciplinary council. For staging, it is first determined whether there is distant organ metastasis (primary metastasis) at the initial diagnosis. If metastatic disease is identified, the treatment plan is altered. According to the National Comprehensive Cancer Network (NCCN) guidelines, the recommended imaging modalities for detecting metastatic disease include thorax and abdominal contrast computerised tomography (CT). A brain MRI is advised in cases where central nervous system symptoms are present. Similarly, vertebral MRI is recommended for detecting vertebral metastases, and positron emission tomography (PET-CT) is suggested for suspected bone metastases [3]. Investigating distant metastases is a costly and time-consuming process [4, 5]. Considering the number of breast cancer patients who are diagnosed annually, it can result in significant time and financial losses.

Although radiological imaging is essential for detecting metastatic disease in breast cancer patients at the time of diagnosis, numerous biomarkers have been studied for the noninvasive detection of metastatic disease in these patients [6–10]. There is currently no high sensitivity and specificity method for detecting distant organ metastasis. Carcinoma Antigen 15 − 3 (CA 15 − 3) is the most studied and notable marker for this condition. CA 15 − 3 has recently been used primarily for follow-up and has no place in breast cancer diagnosis [3].

In this retrospective study, we aimed to evaluate clinicopathological and laboratory data from breast cancer patients to identify distant organ metastases at the initial diagnosis.

## Materials and methods

### Ethical approval and study design

The study was initiated after receiving approval from the institution and the ethics committee (Noninvasive Research Ethics Committee of Fırat University (approval no. 2023/12 − 10)) according to the Declaration of Helsinki. Between January 2021 and January 2022, the electronic data of patients who were subsequently diagnosed with breast cancer were obtained. Written approval was received from Firat University Hospital, where the study was conducted, for the use of the data. The institution requires all patients to give written consent before surgery that includes data use. The inclusion criteria for the study included female patients over 18 years old who were diagnosed with breast cancer, who were evaluated by a multidisciplinary oncology council (medical oncologist, radiologist, and surgical oncologist), and who decided to begin treatment based on the council’s decision. Patients who were pathologically diagnosed with breast cancer for the first time, who had not received any treatment, and who had not undergone metastasis screening previously were included in the study. Standard metastasis screening was performed on all patients, regardless of metastatic symptoms, and additional imaging was performed in all symptomatic patients, as needed or suspected.

### Exclusion criteria

**Fig. 1:**
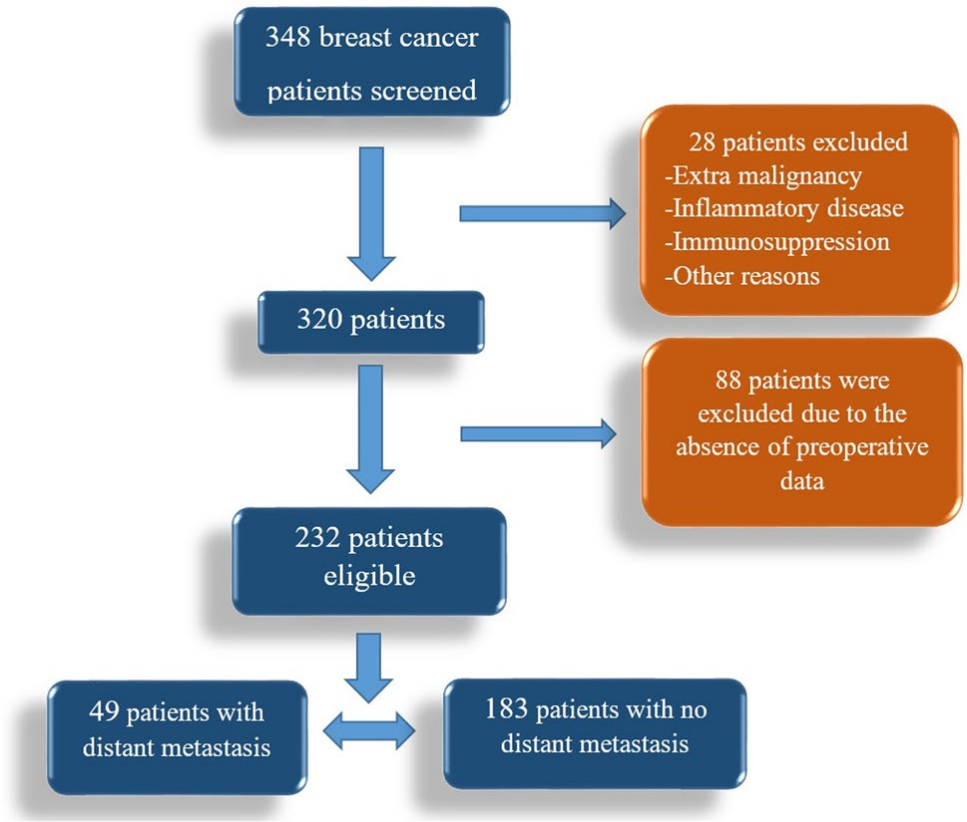
The flow diagram of the cases that were included and excluded

Patients with other systemic cancers, systemic immune diseases, systemic immune therapy, benign ovarian cysts, benign breast disease, benign liver disease, cirrhosis, sarcoidosis, or lupus were excluded. In addition, blood samples collected following surgery or neoadjuvant therapy were excluded. Patients without standard metastasis screening (chest and abdominal tomography) or PET-CT screening data were excluded. Patients with missing data were excluded. Patients were divided into two groups, non-metastatic (Group I) and metastatic (Group II), at the time of initial diagnosis (Fig. 1).

### Laboratory data

The CA 15 − 3, carcinoembryonic antigen (CEA), glucose, alkaline phosphatase (ALP), albumin, calcium, neutrophil, lymphocyte, and platelet levels were obtained. Systemic immune inflammation index (SII) and neutrophil-to-lymphocyte ratio (NLR) were computed (neutrophil*platelet/lymphocyte for SII; neutrophil/lymphocyte for NLR).

The serum levels of the cancer markers CEA and CA 15–3 were determined on an ADVIA Centaur XP analyzer (Siemens Healthineers USA, United States) using the reagent for analyzer Siemens Advia Centaur (Reagement Siemens CA 15 − 3–10327620 and Reagement Siemens CEA – 10309977).

### Pathology records

All pathological assessments are conducted centrally by the institution’s pathologists. T stage is regarded as tumor diameter (T1: ≤2 cm, T2: >2 cm-≤5 cm, T3: >5 cm, and T4 invades skin and/or chest wall or inflammatory cancer).

Axillary lymph node status was evaluated in all patients either clinicoradiologically or by tru-cut biopsy and divided into metastatic or nonmetastatic. N stage is regarded as nonmetastatic (N0) or metastatic to at least one lymph node (N1-3).

### Hormone receptor status

The Olympus Microscope Digital Camera model DP71 (Olympus Co.; Shinjuku, Tokyo, Japan) software imaging system was used for the histological analysis of ER and PR status. *Estrogen(ER)/Progesterone(PR) positivty;* Cytoplasmic staining or < 1% nuclear staining of cells is considered negative and ≥ 1% nuclear staining is considered positive in immunohistochemical staining. Human epidermal growth factor receptor 2(*HER2) staining;* Tumors with immunohistochemical staining of 3+ (uniform, intense membrane staining of 30% of invasive tumor cells) were considered HER2-positive. Cases with an IHC staining of 2 + were considered positive if they turned out to be positive in subsequent HER2/neu gene amplification (fluorescence in situ hybridization). HER2 1 + status and the absence of staining were considered negative. Breast cancer is divided into five molecular luminal types.

### Categorizing luminal types

- Luminal A: Estrogen receptor (ER) positive and progesterone receptor (PR) positive, HER2 negative, and Ki-67 < %14,
- Luminal B Her2(-): ER and/or PR positive, HER2 negative, and Ki-67 ≥ %14,
- Luminal B Her2(+): ER and/or PR positive, HER2 positive,
- HER2-like: ER and PR negative, HER2 positive,
- Triple-negative: ER, PR, and HER2 negative.

### Power analysis

The power analysis was carried out as post-hoc (achieved power) in the G-Power 3.1.9.7 statistics pack (written by Franz Faul, Universität Kiel, Germany) because the predictive usefulness of the CA 15 − 3 on the probability of distant organ metastasis has not been previously studied in the literature. The probability of avoiding a type-II error was defined as having a power of more than 80%. The effect size (w) was 1.04, and the power of the study (1-beta) was 0.95 for predicting distant organ metastasis in breast cancer patients, taking into account the estimated cut-off value at the alpha error probability of 0.05.

### Statistical methods

The normality of the data distribution was assessed using the Kolmogorov-Smirnov and Shapiro-Wilk tests. Parametric data are expressed as mean ± standard deviation (SD) and nonparametric data as median (minimum-maximum). The independent samples t-test was used to compare parametric data, while the Mann-Whitney U and Kruskal-Wallis tests were used to compare nonparametric data. The chi-square or Fisher’s exact test was used to analyze categorical data. The optimal cut-off values for predictor factors were determined using a receiver operating characteristic (ROC) curve. In the ROC analysis, the value with the highest sensitivity and specificity was deemed the cut-off.

## Results

### Total characteristics

A total of 232 female patients were enrolled in the study. The mean age was 52.50 ± 12.64 years. Forty-nine (21.1%) of 232 patients (group I) had metastatic disease. A total of 164 patients (70.7%) had axillary lymph node metastases. The median tumor diameter was 26 mm (2–125). Sixty-five (28%) of the patients had stage T1 disease, 122 (52.6%) had stage T2 disease, 18 (7.8%) had stage T3 disease, and 27 (11.6%) had stage T4 disease. CA 15 − 3 levels, CEA levels, glucose, albumin, ALP, calcium levels, Ki-67, neutrophils, lymphocytes, the SII, the NLR, menopausal status, and pathological data are presented in Table 1. Of the 49 patients with metastatic disease, 27 (11.6%) had metastases to a single organ, while 22 (9.5%) had metastases to multiple organs. Only five patients had a single metastatic lesion.

**Table 1:** Demographic data of patients

Variables		*n* = 232 (100%)
Age (years)		52.50 ± 12.64
Menopausal status	Premenopausal	115 (49.6%)
	Postmenopausal	117 (50.4%)
CA 15 − 3 (U/mL)		19.75 (2.8–1841)
CEA (ng/mL)		1.37 (0.01–303)
Glucose (mg/dL)		100 (71–321)
ALP (U/L)		77 (25–337)
Albumin (g/dL)		4.3 (2.9–5.9)
Calcium (mg/dL)		9.4 (6–11)
Neutrophil (10e3/µL)		4.28 (0.51–10.47)
Lymphocyte (10e3/µL)		1.84 (0.18–4.42)
Platelet (10e3/µL)		283 (110–672)
SII		671.57 (81.05–3331.48.05.48)
NLR		2.38 (0.59–13.50)
Breast side	Right	120 (51.7%)
	Left	105 (45.3%)
	Bilateral	7 (3%)
Tumor histology	IDC	207 (89.2%)
	ILC	19 (8.2%)
	Mixt type	6 (2.6%)
Tumor diameter (mm)		26 (2–125)
T-stage	I	65 (28%)
	II	122 (52.6%)
	III	18 (7.8%)
	IV	27 (11.6%)
N-stage	N 0	68 (29.3%)
	N (+)	164 (70.7%)
M stage	M 0	183 (78.9%)
	M 1	49 (21.1%)
ER	ER -	62 (26.7%)
	ER +	170 (73.3%)
PR	PR -	99 (42.7%)
	PR +	133 (57.3%)
HER2	HER2 -	151 (65.1%)
	HER2 +	81 (34.9%)
Ki-67 (%)		30 (1–95)
Luminal type	Luminal A	34 (14.7%)
	Luminal B HER2 (-)	88 (37.9%)
	Luminal B HER2 (+)	62 (26.7%)
	HER2 like	19 (8.2%)
	Triple-negative	29 (12.5%)
Bone metastasis	None	200 (90.9%)
	Metastasis	32 (9.1%)
Lung metastasis	None	211 (90.9%)
	Metastasis	21 (9.1%)
Liver metastasis	None	217 (93.5%)
	Metastasis	15 (6.5%)
Brain metastasis	None	226 (97.4%)
	Metastasis	6 (2.6%)
Other metastasis	None	227 (97.8%)
	Metastasis	5 (2.2%)
Single/2 or more organ metastasis	None	183 (78.9%)
	Single organ	27 (11.6%)
	2 or more organ	22 (9.5%)
Metastatic lesions	None	183 (78.9%)
	Single lesion	5 (2.2%)
	Multiple lesions	44 (19%)

### Comparing the groups

There was a significant difference between the groups in CA15-3, CEA, albumin, ALP, neutrophil count, lymphocyte count, tumor diameter, NLR, SII, T stage, and N stage (*p* < 0.05). Glucose, calcium, platelet, Ki-67, menopausal status, breast side, tumor histological type, ER, PR, HER2 receptor status, and luminal type were not significantly different (*p* > 0.05) (Table 2).

**Table 2:** Differences between metastatic vs. nonmetastatic patients

Variables			Group I *n* = 183	Group II *n* = 49	*p* value
Age (years)			51.58 ± 11.85	57.21 ± 14.22	0.064
CA 15 − 3 (U/mL)			17.5 (2.8–313.7.8.7)	60.4 (26.9–1841)	< 0.001
CEA (ng/mL)			1,23 (0.01–138)	2.5 (0.01–303)	< 0.001
Glucose (mg/dL)			100 (71–321)	104 (71–312)	0.094
ALP (U/L)			76 (25–196)	94 (48–337)	0.001
Albumin (g/dL)			4.30 (3.4–5.4)	4.1 (2.5–4.9)	< 0.001
Calcium (mg/dL)			9.4 (6.48–10.6)	9.4 (6–11)	0.777
Neutrophil (10e3/µL)			4.06 (1.4–10.4)	5.17 (0.51–10.47)	0.001
Lymphocyte (10e3/µL)			1.93 (0.4–4.42)	1.56 (0.18–3.1)	0.002
Platelet (10e3/µL)			283 (110–613)	288(138–672)	0.809
NLR			2.15 (0.59–13.5)	3.25 (0.72–7.59)	< 0.001
SII			639.99 (81.05–2708.18.05.18)	864.64 (111.87–3331.48.87.48)	0.001
Tumor diameter			25 (2–125)	28 (10–109)	0.036
Ki-67 (%)			28 (1–95)	30 (2–90)	0.095
Menopausal status		Premenopausal	94	21	0.290
		Postmenopausal	89	28	
ER		ER +	132	38	0.446
		ER -	51	11	
PR		PR +	105	28	0.977
		PR -	78	21	
HER2		HER2 +	66	15	0.477
		HER2 -	117	34	
Luminal types					0.875
	Luminal A		28	6	
	Luminal B HER2 -		66	22	
	Luminal B HER2 +		50	12	
	HER2 like		16	3	
	Triple-negative		23	6	
T stage		I	58	7	0.001
		II	99	23	
		III	12	6	
		IV	14	13	
N stage		N 0	63	5	0.001
		N (+)	120	44	
Tumor histology		IDC	162	45	0.641
		ILC	15	4	
		Mixt type	6	0	
Breast side		Left	87	18	0.144
		Right	92	28	
		Bilateral	4	3	

### ROC analysis

**Fig. 2:**
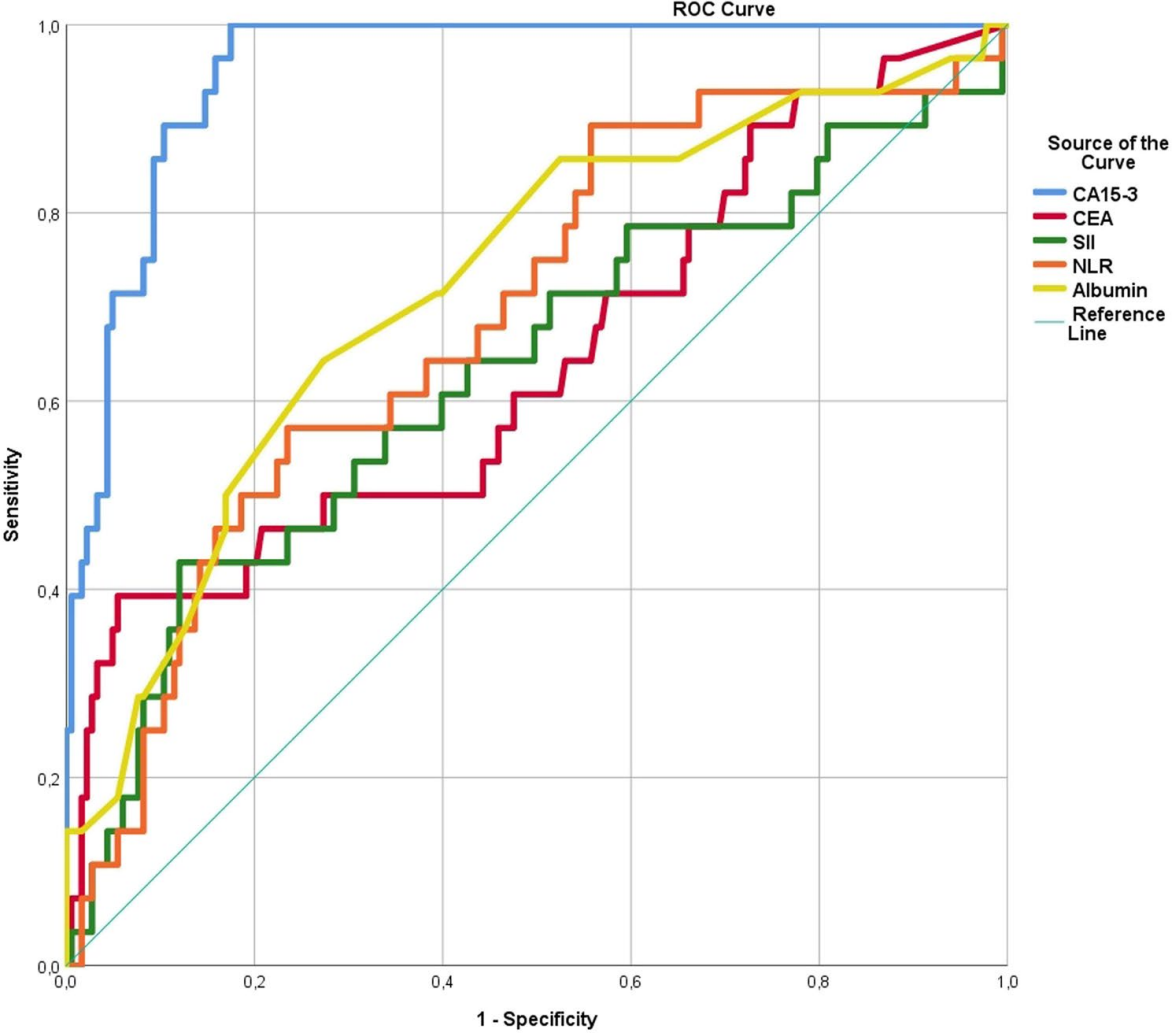
ROC curves of CA 15 − 3, CEA, albumin, SII, and NLR levels

ROC curve analysis was used to determine the discrimination ability of CA 15 − 3, CEA, albumin, NLR, and SII levels in detecting distant metastasis. The levels of CA 15 − 3, CEA, albumin, NLR, and SII were significantly different (Table 3). Cut-off values for the ability to discriminate were calculated as follows: 25.8 for CA 15 − 3, 2.36 for CEA, 4.04 for albumin, 2.71 for NLR, and 1053.62 for SII (Fig. 2). When the data were dichotomized by applying cut-off values, all 151 (65.1%) patients with CA 15 − 3 values below 25.8 were determined to be non-metastatic. Among the other 81 (34.9%) patients above the cut-off value, 49 had metastatic disease, and 32 had non-metastatic disease (Table 4). With a value of 25.8, the sensitivity of CA15-3 in predicting metastatic disease was 100%, the specificity was 82.5%, the positive predictive value (PPV) was 60.5%, and the negative predictive value (NPV) was 100%. A total of 65.1% of patients were determined to be nonmetastatic based on the current CA15-3 cutoff value.

**Table 3:** AUC-ROC and cut-off values of variables

Variables	AUC – 95% CI	*p* value	Cut-off value
CA 15 − 3 (U/mL)	*0.950* (0.924–0.975)	< 0.001	25.8
CEA (ng/mL)	0.692 (0.603–0.781)	< 0.001	2.36
Albumin (g/dL)	0.661 (0.567–0.755)	< 0.001	4.04
NLR	0.707 (0.623–0.790)	< 0.001	2.71
SII	0.661 (0.567–0.755)	< 0.001	1053.62

**Table 4:** Sensitivity, specificity, PPV, and NPV of variables at their cut-off values for metastasis prediction

Variables		Group I *n* = 183	Group II *n* = 49		
CA 15 − 3 (U/mL)	< 25.8	151	0	Sensitivity	100%
				Specificity	82.5%
	≥ 25.8	32	49	PPV	60.5%
				NPV	100%
CEA (ng/mL)	< 2.36	144	22	Sensitivity	55.1%
				Specificity	78.69%
	≥ 2.36	39	27	PPV	40.9%
				NPV	86.7%
Albumin (g/dL)	< 4.04	31	22	Sensitivity	44.9%
				Specificity	83.1%
	≥ 4.04	152	27	PPV	41.5%
				NPV	84.9%
NLR	< 2.71	134	18	Sensitivity	63.27%
				Specificity	73.22%
	≥ 2.71	49	31	PPV	38.8%
				NPV	88.2%
SII	< 1053.62	157	26	Sensitivity	46.9%
				Specificity	85.8%
	≥ 1053.62	26	23	PPV	46.9%
				NPV	85.8%

Using a CA 15 − 3 cutoff value of 25.8, a statistically significant difference was observed between the two groups in terms of T and N stages, and levels of CEA, serum albumin, glucose, ALP levels, and lymphocyte-neutrophil counts, the SII, and the NLR (*p* < 0.05) (Table 5). There was no statistically significant difference between the groups with high and low CA15-3 levels regarding hormone receptor status, luminal types, menopausal status, breast side, tumor histology, or age (*p* > 0.05).

**Table 5:** Differences between high and low CA15-3 levels according to the cut-off value

Variables		CA 15 − 3 < 25.8 *n* = 151	CA 15 − 3 ≥ 25.8 *n* = 81	*p* value
Age (years)		51.25 ± 11.98	54.83 ± 13.56	0.096
CEA (ng/mL)		1.2 (0.01–138)	2.08 (0.01–303)	< 0.001
Glucose (mg/dL)		99 (71–321)	105 (71–312)	0.049
ALP (U/L)		75.5 (25–196)	86 (28–337)	0.002
Albumin (g/dL)		4.3 (3.4–5.4)	4.2 (2.5–4.9)	0.010
Calcium (mg/dL)		9.4 (6.48–10.60)	9.45 (6–11)	0.989
Neutrophil (10e3/µL)		4.06 (1.40–10.40)	4.76 (0.51–10.47)	0.025
Lymphocyte (10e3/µL)		1.97 (0.40–4.42)	1.60 (0.18–3.16)	0.001
Platelet (10e3/µL)		280 (110–613)	291 (138–672)	0.360
SII		606.86 (81.05–2708.18.05.18)	801.80 (111.87–3331.48.87.48)	< 0.001
NLR		2.23 (0.59–13.50)	2.72 (0.72–7.59)	< 0.000
Ki-67 (%)		25 (1–95)	30 (1–90)	0.053
T-stage	I	52	13	0.001
	II	79	43	
	III	9	9	
	VI	11	16	
N-stage	N(0)	51	17	0.041
	N(+)	100	64	
ER	Positive	115	55	0.175
	Negative	36	26	
PR	Positive	89	44	0.498
	Negative	62	37	
HER2	Positive	52	29	0.835
	Negative	99	52	
Luminal types	Luminal A	26	8	0.543
	Luminal B HER2 (-)	56	32	
	Luminal B HER2 (+)	41	21	
	HER2 like	11	8	
	Triple negative	17	12	
Menopausal status	Premenopausal	81	34	0.090
	Postmenopausal	70	47	
Breast side	Left	71	34	0.128
	Right	78	42	
	Bilateral	2	5	
Tumor histology	IDC	133	76	0.725
	ILC	13	6	
	Mixt type	5	1	

## Discussion

This study retrospectively analyzed the demographic, laboratory, and histopathological data of breast cancer patients who presented with distant metastases at the time of initial diagnosis. Although the effectiveness of CA 15 − 3 in detecting metastatic patients is unsatisfactory, its efficacy in detecting nonmetastatic patients can be useful. No patient whose CA 15 − 3 level was less than 25.8 U/mL was found to have distant organ metastasis. This seems possible only for patients with low CA15-3 levels in the future. With other data, significant results have been obtained, but they cannot yet be implemented clinically to detect metastatic disease.

CA 15 − 3 is derived from Mucin Short Variant S1 (MUC1). MUC1, also called polymorphic epithelial mucin (PEM) or epithelial membrane antigen (EMA), is a mucin encoded by the MUC1 gene in humans [11]. Although CA 15 − 3 is used in breast cancer patients during follow-up, the American Society of Clinical Oncology (ASCO) and NCCN guidelines do not recommend it for screening, diagnosis, or follow-up after primary treatment [3, 12]. Recent studies have demonstrated a correlation between CA 15 − 3 levels and overall survival (OS) and/or disease-free survival in patients with breast cancer [13–16]. According to previous studies, axillary nodal status and distant metastasis were correlated with CA 15 − 3 levels [7, 9, 17]. At the time of the initial diagnosis of breast cancer, prior to receiving any treatment, CA 15 − 3 levels were higher in patients with distant metastases than in those without metastases [11]. The ability of CA15-3 to detect breast cancer metastasis (both axillary and distant organs combined) was studied by El-mezayen et al. They reported the AUC and cutoff values, but no data were given for patients with distant metastases only [7].

In a study of distant metastasis in breast cancer, the CA 15 − 3 levels were found to be high in metastatic patients. Patients with CA 15 − 3 > 30 U/L were included in this study [18]. Thus, its ability to discriminate between metastatic diseases is limited. In addition, distinct cut-off values were reported for hormone receptors. In our study, we included all patients whose CA 15 − 3 levels were evaluated, and no significant differences between luminal types were observed. This may be due to the limited number of patients.

Research on metastatic breast cancer has predominantly focused on investigating its association with receptor status [10, 19]. Prognostic studies have been conducted for CA 15 − 3, and it has been highlighted that CA 15 − 3 may be an early recurrence or metastasis indicator. In recent studies, CA 15 − 3 levels have been known to be elevated in cases of primary metastasis [9, 18]. Wang et al. studied additional breast cancer biomarkers, including CA 15− 3, for metastatic breast cancer [6]. At the cutoff value of 14.96 U/mL, they indicated that CA 15 − 3 is not a useful marker for metastasis prediction. With the combination of these markers, they were able to achieve greater sensitivity and specificity. The highest AUC and Youden index were observed when combining CEA and tissue polypeptide-specific antigen. However, whether the patients included in the study were metastatic at the time of their initial diagnosis or secondary metastatic patients was not specified. Essentially, it appears that patients with secondary metastatic disease were enrolled in the study. Therefore, the diagnostic value of CA15-3 appears to be low. Our study included only metastatic breast cancers that had not received any treatment at the time of initial diagnosis, and patients with secondary metastases were excluded.

CEA is also a marker commonly used in postoperative monitoring. It is utilized for treatment response evaluation, early diagnosis of metastasis, and recurrence detection [6, 13, 20]. No studies that used CEA for distant organ metastasis at the time of breast cancer diagnosis could be found. In our study, even though CEA varied significantly between groups, it demonstrated low sensitivity.

In cancer patients, an energy deficit caused by increased metabolism can result in hypoalbuminemia. This can occur in breast cancers as well [21, 22]. We are unable to comment due to the lack of research on albumin concentration and distant organ metastasis. In our study, there was a statistically significant difference in the albumin concentration between groups, and albumin concentration had a low degree of sensitivity and specificity.

SII and NLR, which are used to predict OS and disease-free survival in breast cancer patients, have not previously been studied in primary metastasis [23, 24]. Both markers were significantly different between the groups in the present study. Due to their low sensitivity, their clinical use is difficult.

Imaging for metastasis is a standard procedure in patients diagnosed with breast cancer. NCCN recommends thoracoabdominal CT or PET-CT for this purpose [3]. In symptomatic patients, it is recommended to perform additional vertebral or brain MRI. In other words, standard MRI imaging is not performed on all patients. In our study, CA 15 − 3 levels were found to be above the cut-off in patients with metastasis detected by MRI. So, according to our study, this patient group was not missed.

Our study is limited by the small number of patients with metastatic disease and the lack of a standardized detection method. The disproportionate distribution between the metastatic and non-metastatic categories may introduce bias in the data. This disproportionality is due to the low number of patients with metastatic breast cancer at the time of initial diagnosis. In every patient, chest and abdomen tomography or PET-CT is used to detect metastatic disease. MRI was used to diagnose bone or brain metastases in symptomatic patients. Another limitation is that CA 15 − 3 requires validation to confirm its ability to distinguish metastatic disease.

In conclusion, metastatic versus non-metastatic breast cancer patients showed differences in terms of T-stage, N-stage, and levels of CA 15 − 3, CEA, albumin, ALP, NLR, and SII. Although imaging is the gold standard, the CA 15 − 3 value at the time of diagnosis in patients with breast cancer could be used to differentiate between distant metastasis and non-metastatic patients. In this manner, reducing both the time loss and the high health care costs caused by routine tomography or other imaging techniques for patients may be possible. This seems possible only for patients with low CA 15 − 3 levels in the future. New prospective studies are required to confirm this circumstance. This is the first study to examine the ability of CA 15 − 3 to differentiate between metastatic and non-metastatic breast cancer patients at initial diagnosis.

## Data Availability

No datasets were generated or analysed during the current study.

## Abbreviations

CA 15-3: Cancer antigen 15-3
CEA: Carcinoembryonic antigen
ROC: Receiver operating characteristic
AUC: Area under the curve
SII: Systemic immune-inflammation index
NLR: Neutrophil lymphocyte ratio
ALP: Alkaline phosphatase
PET-CT: Positron emission tomography–computed tomography
IHC: Immunohistochemistry
ER: Estrogen
PR: Progesterone
HER2: Human epidermal growth factor receptor 2
PPV: Positive predictive value
NPV: Negative predictive value
